# Spectrum of Orbital Cellulitis on Magnetic Resonance Imaging

**DOI:** 10.7759/cureus.9663

**Published:** 2020-08-11

**Authors:** Ruchir Jyani, Dilip Ranade, Priscilla Joshi

**Affiliations:** 1 Radiology, Bharati Vidyapeeth Hospital, Pune, IND

**Keywords:** orbital, cellulitis, abscess, magnetic resonance imaging, intracranial complications, cavernous sinus thrombophlebitis, thrombosis

## Abstract

Introduction

Orbital infection is an ophthalmological emergency as it can lead to blindness and intracranial spread. Imaging is needed to determine the extent of the infection, to localize an abscess, and for surgical planning. The role of magnetic resonance imaging (MRI) is well established in the evaluation of orbital pathologies, including orbital cellulitis and abscess, mainly due to its ability to evaluate early intracranial involvement. The objective of the study was to highlight the spectrum of MR imaging findings and the pattern of spread in fifteen patients with orbital cellulitis.

Methods

A prospective study was conducted in a tertiary care hospital. Fifteen patients of all age groups, of either sex, presenting with clinical findings suggestive of orbital cellulitis, referred for MRI of orbits, were included in the study. Written informed consent was obtained prior to the study. Patients’ demographic data such as age and gender, associated co-morbidities, complications, and the pattern of spread of disease on MRI were recorded and evaluated. Descriptive statistics were used.

Results

Orbital/periorbital abscess was found to be the most common complication of orbital cellulitis (eight cases, 53.3%), followed by optic neuritis/perineuritis (four cases, 26.67%), intracranial involvement (four cases, 26.67%), dacryoadenitis (three cases, 20%) and cavernous sinus thrombophlebitis (three cases, 20%). Seven cases (46.67%) had right orbital involvement. Sinusitis was found to be the most common predisposing factor. Amongst the cases associated with sinusitis, the commonest inflamed paranasal sinus was found to be the ethmoid sinus (twelve cases). Amongst the fifteen cases of orbital/periorbital cellulitis, there were only two cases of isolated preseptal cellulitis (13.33%), five cases of postseptal cellulitis (33.33%) and eight cases of both preseptal and postseptal orbital cellulitis (53.33%).

Conclusion

MRI is the imaging modality of choice in the evaluation of orbital cellulitis because of its superior soft tissue and contrast resolution. It is vital to evaluate the extent of the orbital infection, underlying paranasal sinus involvement, as well as detect complications of orbital cellulitis, especially intracranial spread.

## Introduction

Orbital cellulitis is one of the most common orbital pathologies which can be either preseptal (periorbital) or postseptal (orbital). The fibrous orbital septum normally does not allow the spread of periorbital infections into the orbit.

Orbital infection is most often caused by direct extension from adjacent structures; hematogenous infection is less common. The differentiation between periorbital and orbital infections is important clinically because their management is different. Preseptal or extraconal infections are treated by standard antimicrobial therapy. Postseptal infections have a higher rate of complications like intracranial spread, meningitis, and cavernous sinus thrombosis, which can be potentially life-threatening, therefore, they require early detection by MRI and subsequent aggressive management. Identification of orbital abscesses is also important because they may require surgical intervention [1].

Orbital cellulitis causes diffuse, oedematous infiltration of the orbital connective tissue that is best demonstrated by the high signal intensity in T2-weighted fat-saturated sequences. Other findings are swelling and ill-defined margins of the extraocular muscles and exophthalmos [2]. Orbital cellulitis may be complicated by an abscess, which may form in the extraconal or intraconal orbit separate from the bone [3].

Periorbital cellulitis is a preseptal process, which is limited to the soft tissues anterior to the orbital septum. It usually occurs due to the contiguous spread of infection from adjacent structures such as the teeth and face. Computed tomography (CT) and MRI demonstrate diffuse soft-tissue thickening anterior to the orbital septum. Periorbital cellulitis is treated with standard antibiotics on an outpatient basis [4]. Infection in orbit, whether as a result of periorbital cellulitis extending across the orbital septum or due to sinusitis, constitutes an emergency.

Imaging is imperative in the evaluation of the orbit in conjunction with physical examination and fundoscopy for the diagnosis of retro-orbital lesions [5]. MRI is better than CT owing to its increased slice orientation as well as superior anatomic (T1-weighted) and pathologic (T2-weighted [T2W]) resolution. MRI provides excellent contrast resolution in the orbit with the demonstration of pathologies in the intraconal and extraconal compartments. The ability to depict cross-sectional anatomy and pathology with better tissue characterization and even without administering intravenous gadolinium-based contrast agent is a distinct advantage of MRI over CT scanning. In addition, no ionizing radiation is involved in MRI, making the study particularly useful in pregnancy and pediatric population. The multiplanar imaging capability and superior contrast resolution of MRI allow a more effective demonstration of lesions and can be beneficial for surgical planning [6].

Contrast administration is imperative to distinguish an abscess from a phlegmon and edema. The center of the abscess shows restriction of diffusion on diffusion-weighted imaging (DWI) and demonstration of this finding may obviate the need for intravenous contrast administration in patients who have a contraindication to contrast especially the diabetics who commonly have associated nephropathy. MRI has a high sensitivity in the detection of cavernous sinus thrombosis, which is an uncommon but serious complication of orbital cellulitis and paranasal sinusitis. T2W fat-suppressed sequence is especially useful for the detection of edema related to orbital infections. Poor bony detail, susceptibility to artifacts, lack of wider availability, and higher costs remain disadvantages of MRI.

Paranasal sinusitis is the commonest cause of orbital cellulitis and is the underlying cause in two-thirds of all cases. The orbital foreign body is the etiology in approximately 25% of the cases, while the remaining 8-10% cases result from the extension of facial skin infections [7]. Fungal infections frequently start in the paranasal sinuses and nasal cavities and extend into the orbits, especially the orbital apex. The radiologic findings consist of hyperdense contents or non-specific soft tissue densities, frequently associated with bone destruction [8].

## Materials and methods

The study was conducted over a period of two years. It was approved by the ethics committee. Patients with signs and symptoms of orbital cellulitis were included in the study. Patients with isolated involvement of eyelid or conjunctiva without intraorbital extensions, patients with orbital trauma, patients having iron and other magnetic foreign bodies in orbit and those with general MRI contraindications were excluded from the study.

Patients’ demographic data such as age and gender, associated co-morbidities, complications, and the pattern of spread of disease on MRI were recorded and evaluated. Descriptive statistics were used. 

The following approach was used:

1) Written informed consent was taken from the participants. 

2) A detailed history was noted. Any previous radiological investigations were also asked for, and obtained, if possible, for comparison as well as aid to reporting. 

3) As and when required, in uncooperative patients, sedation was given, for which patients would be kept nil by mouth for a minimum of four hours. 

4) Imaging characteristics of orbital lesions were evaluated and correlated with the culture or postoperative findings wherever available. 

MRI was performed on a 1.5 Tesla MR scanner (Philips Achieva) using a 16 channel Neurovascular (NV)-16 coil. The gradient strength of the magnet was 33 mT/m.

Multiplanar, multi-echo MRI of the orbits was performed with the following sequences, as given in Table 1.

**Table 1:** Protocol used in MRI orbits STIR - short tau inversion recovery

Plane	Sequences				
Axial	T1	T2		T1 fat-saturated pre-contrast	T1 fat-saturated post-contrast
Coronal	T1	T2	STIR	T1 fat-saturated pre-contrast	T1 fat-saturated post-contrast
Sagittal oblique			STIR	T1 fat-saturated pre-contrast	T1 fat-saturated post-contrast

The parameters used are tabulated in Table 2.

**Table 2:** Parameters used in MRI orbits STIR - short tau inversion recovery; TR - repetition time; TE - echo time

	TR (ms)	TE (ms)	Matrix	Slice thickness	Slice gap	Flip angle
T1	450	15	256 x 150	3mm	1mm	90
T2	6035	100	256 x 150	3mm	1mm	90
STIR	4045	90	256 x 150	3mm	1mm	90

## Results

**Figure 1:**
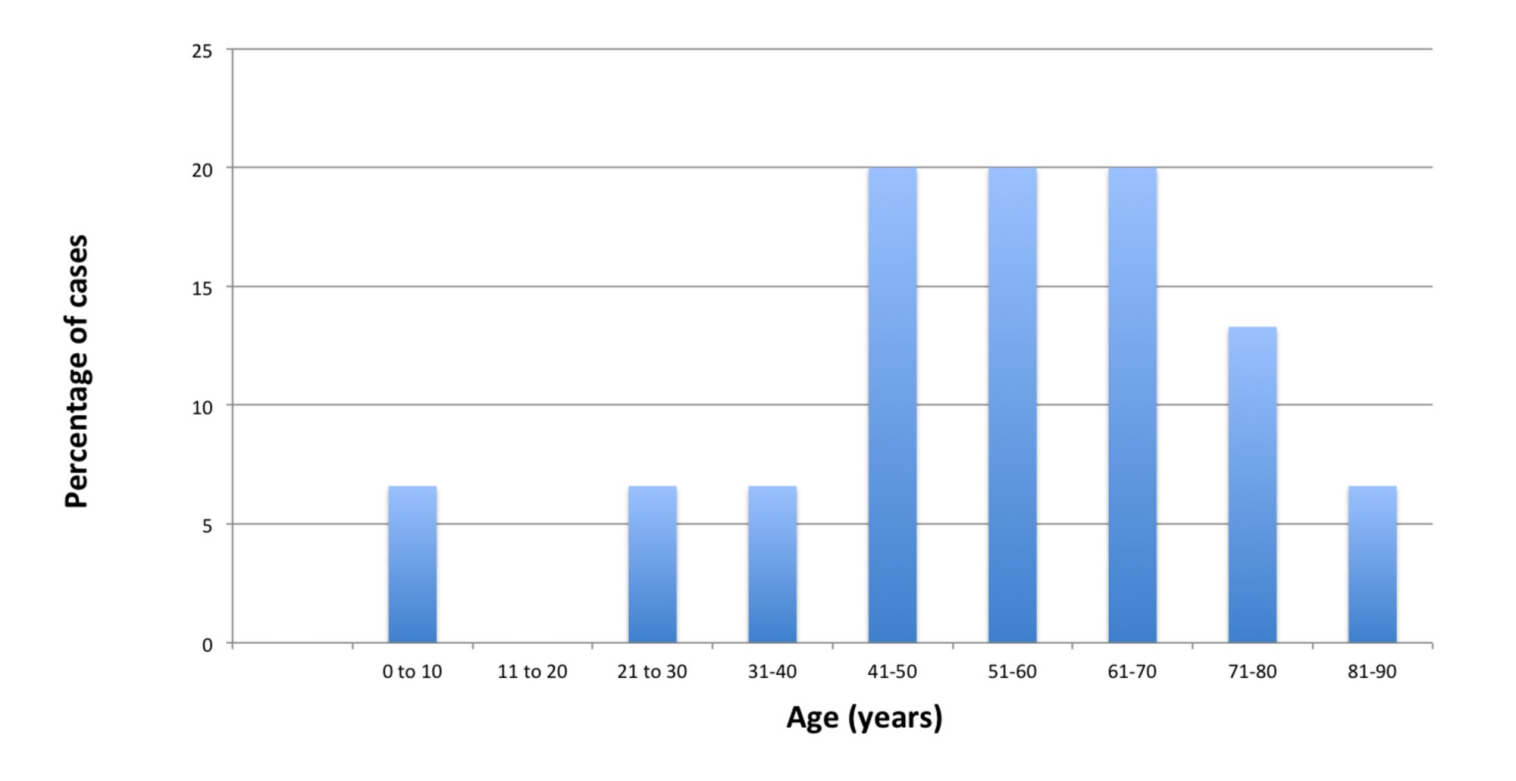
Graph showing the age distribution of fifteen cases of orbital cellulitis

In the present study, the maximum number of patients i.e., three each (20%), were found in the age group of 41 to 50 years, 51 to 60 years, and 61 to 70 years (Figure 1). 

Males (10, 66.67%) were more commonly affected than females (5, 33.33%), with male to female ratio being 2:1. The gender distribution is depicted in Table 3.

**Table 3:** Gender wise distribution of patients.

Gender	Number of patients	Percentage (%)
Male	10	66.67%
Female	5	33.33%
Total	15	100%

The demographic data and co-morbid conditions are illustrated in Table 4.

**Table 4:** Demographics and co-morbid conditions F - frontal; E - ethmoid; M - maxillary; S - sphenoid

Case No.	Age (years) / sex	Diabetes	Skin Infection	Sinusitis
1.	62 / M	Yes	No	E, M, S
2.	44 / F	No	No	F, E, M, S
3.	48 / F	No	Yes	None
4.	51 / M	No	Yes	F, E, M, S
5.	60 / M	No	No	E, M, S
6.	32 / M	No	No	E, M
7.	71 / M	No	No	E, S
8.	66 / F	No	Yes	M, S
9.	71 / F	No	No	F, E, M
10.	58 / F	No	Yes	E, M, S
11.	85 / M	Yes	Yes	F, E
12.	63 / M	No	No	E, M
13.	21 / M	No	No	None
14.	2 / M	No	No	E, M
15.	45 / M	No	Yes	E

Out of fifteen patients with orbital cellulitis, seven (46.67%) had right orbital involvement, seven (46.67%) had left orbital involvement, and one patient (6.67%) had bilateral involvement. 

Twelve cases (80%) showed involvement of multiple paranasal sinuses and amongst the cases associated with sinusitis, the commonest inflamed paranasal sinus was found to be the ethmoid sinus (twelve cases), followed by the frontal sinus (four cases). None of the sinuses were involved in two cases, whereas one case (6.6%) showed the involvement of single (ethmoid) sinus.

Amongst the fifteen cases of orbital/periorbital cellulitis, there were only two cases of isolated preseptal cellulitis (13.33%), five cases of postseptal cellulitis (33.33%) and eight cases of both preseptal and postseptal orbital cellulitis (53.33%).

**Figure 2:**
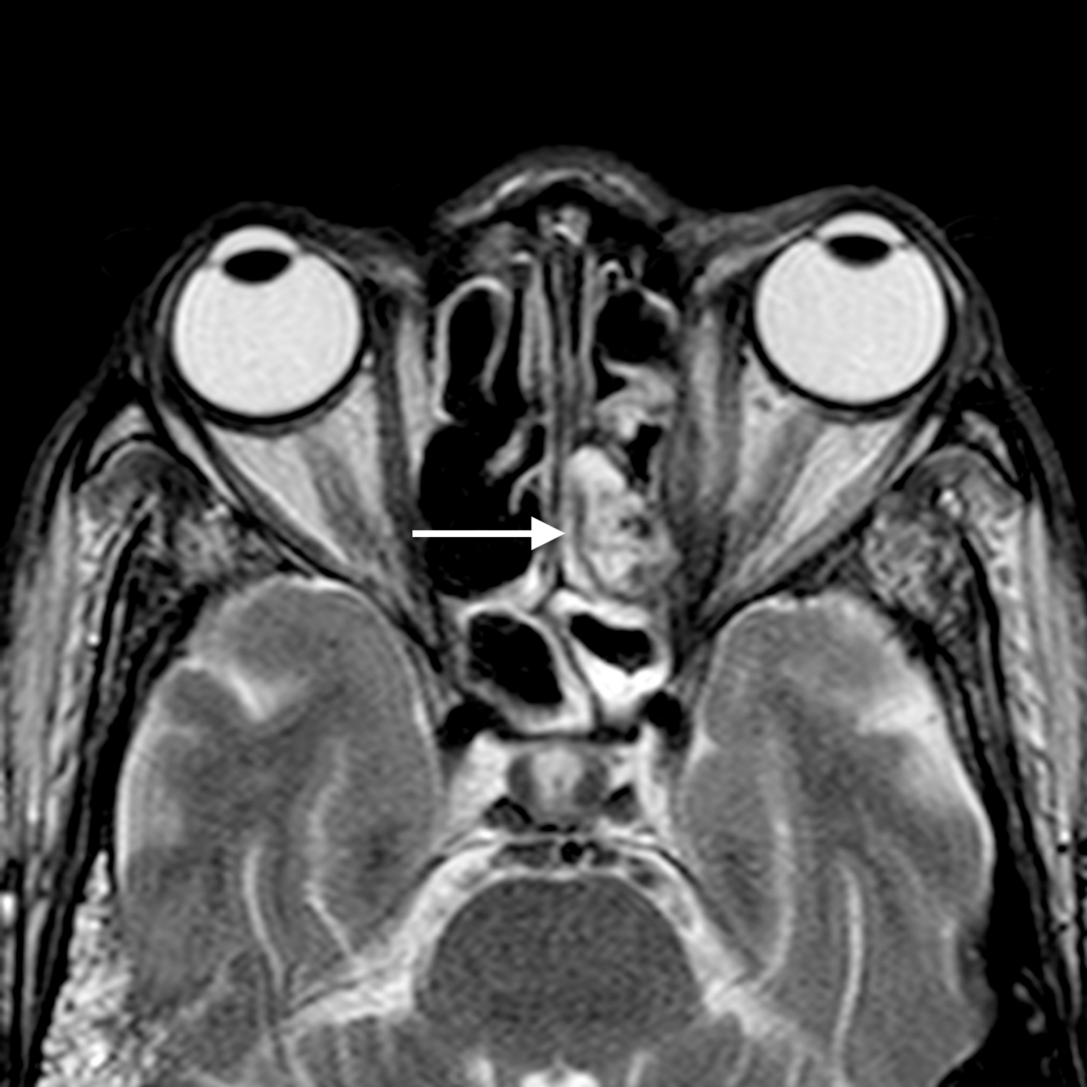
Orbital cellulitis: axial T2WI shows abnormal soft tissue in the left posterior ethmoid air cells (arrow) appearing hyperintense TW2I - T2-weighted image

**Figure 3:**
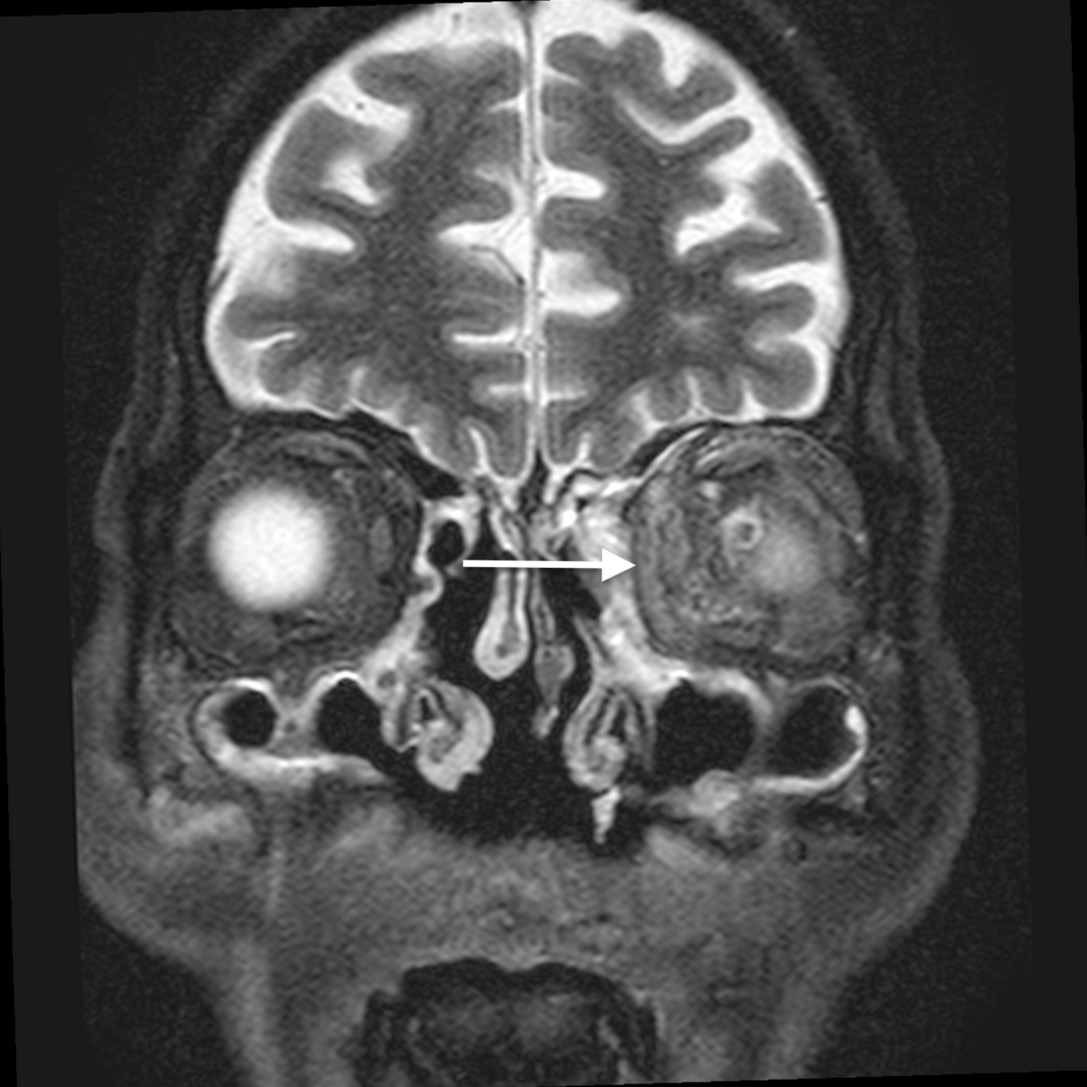
Orbital cellulitis: coronal STIR image shows hyperintense soft tissue (arrow) extending into the extraconal compartment of the left orbit, abutting the medial rectus with surrounding fat stranding STIR - short tau inversion recovery

**Figure 4:**
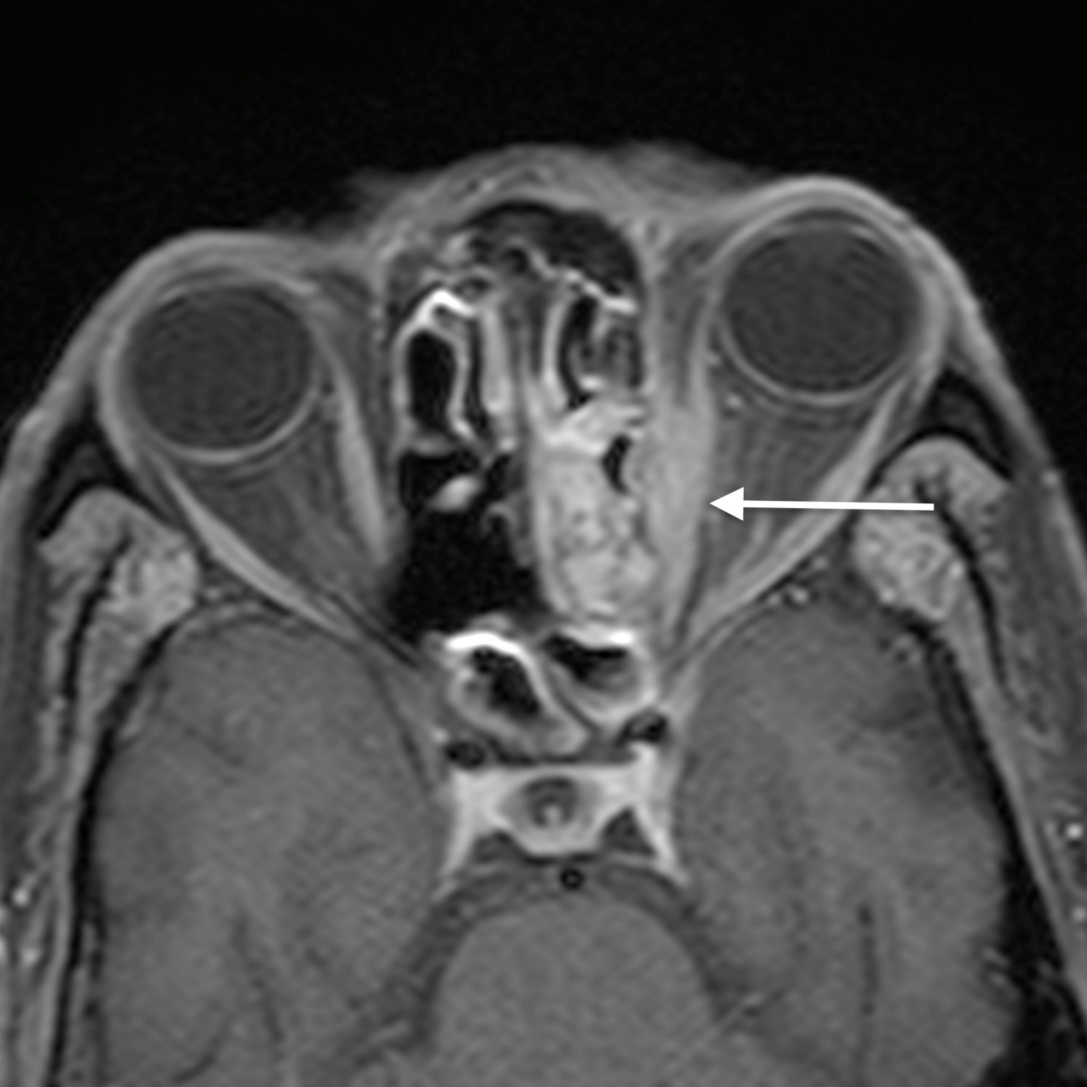
Orbital cellulitis: axial post-contrast T1WI shows enhancement of the abnormal soft tissue (arrow) T1WI - T1-weighted image

**Figure 5:**
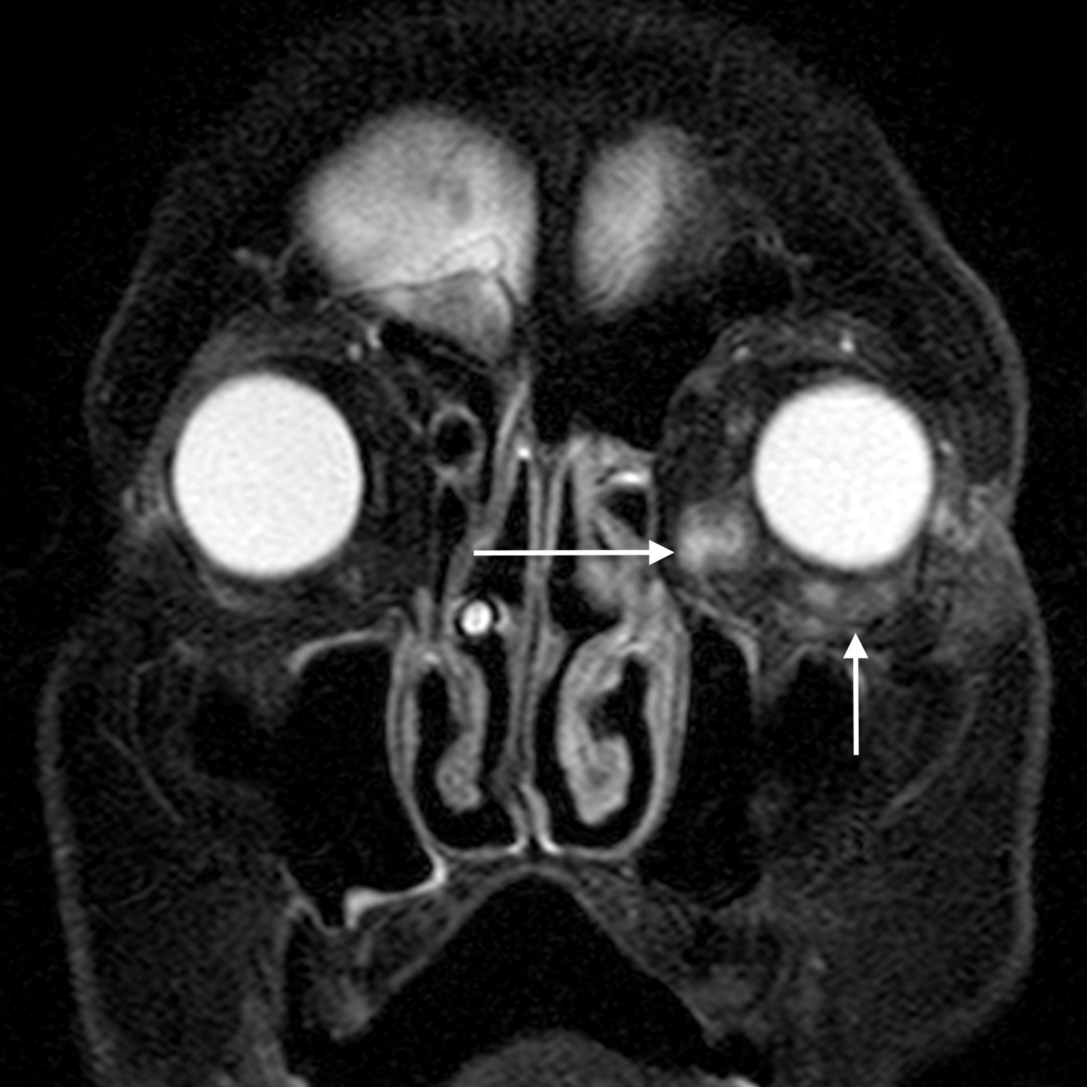
Orbital abscess: coronal STIR image shows two well defined hyperintense lesions (arrows) in the intraconal and extraconal compartments of left orbit in the inferior and medial aspects STIR - short tau inversion recovery

**Figure 6:**
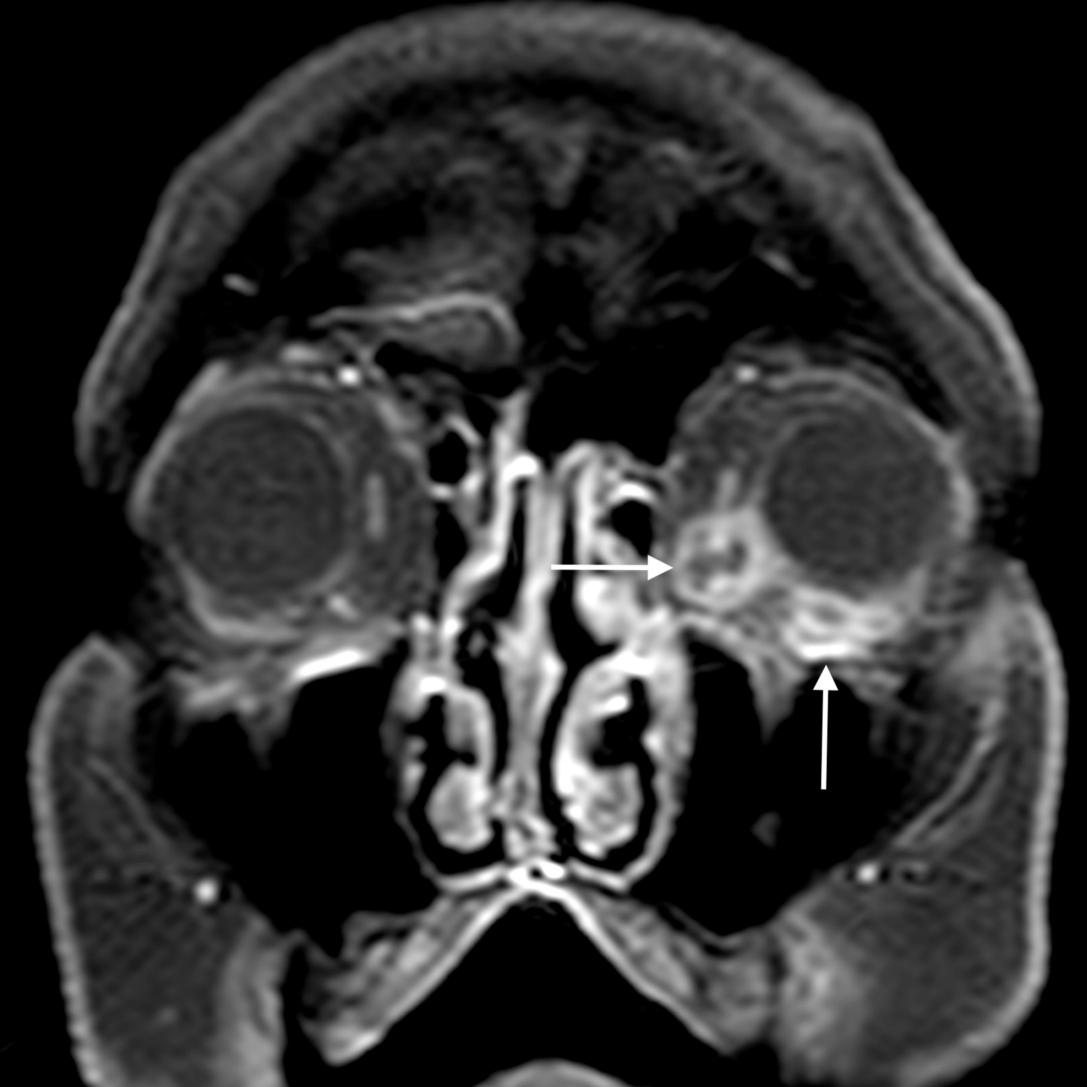
Orbital abscess: coronal post gadolinium T1WI shows peripheral enhancement of the lesions (arrows), indicative of abscess T1WI - T1-weighted image

**Figure 7:**
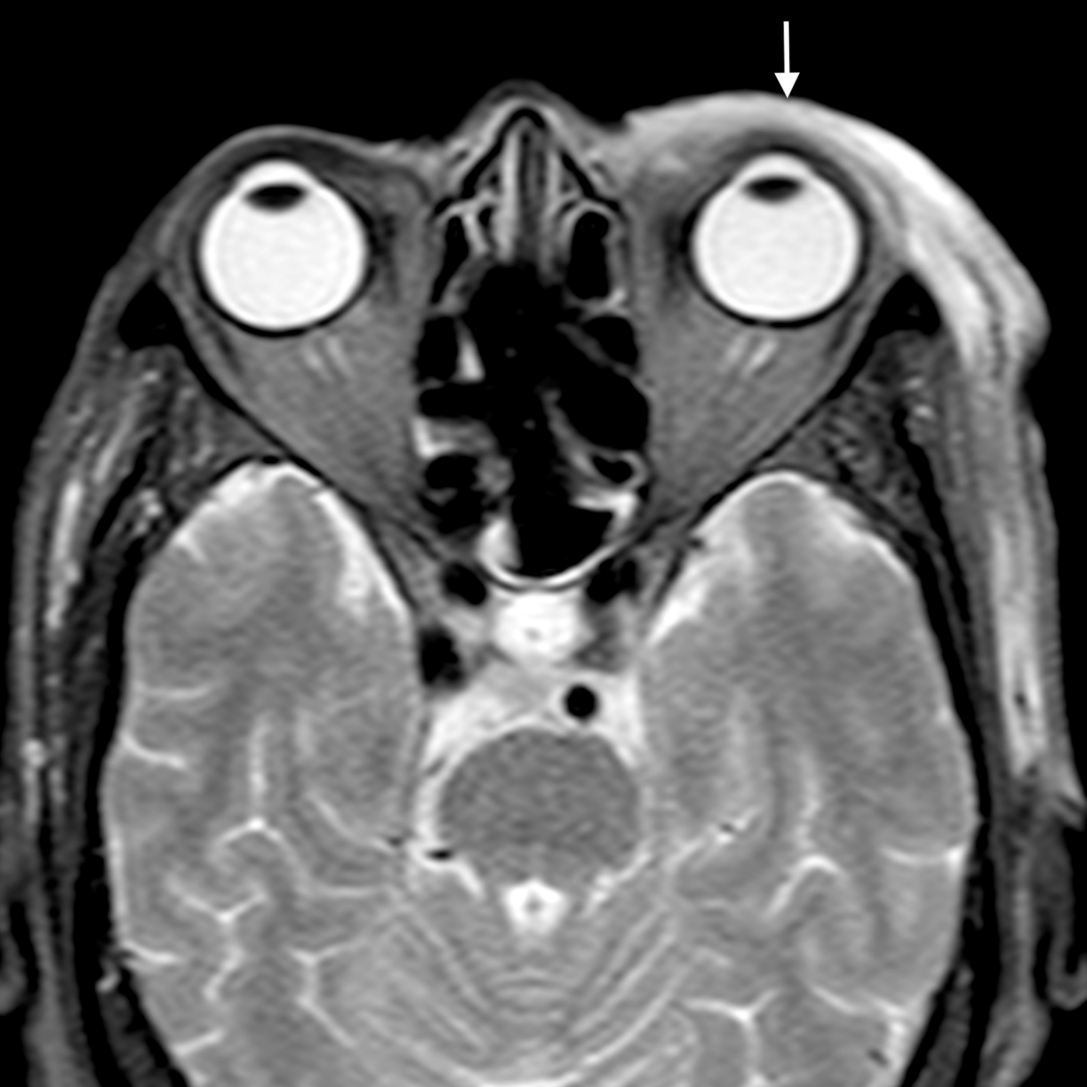
Preseptal cellulitis with abscess: axial STIR image shows abnormal hyperintense signal in the preseptal region in subcutaneous plane STIR - short tau inversion recovery

**Figure 8:**
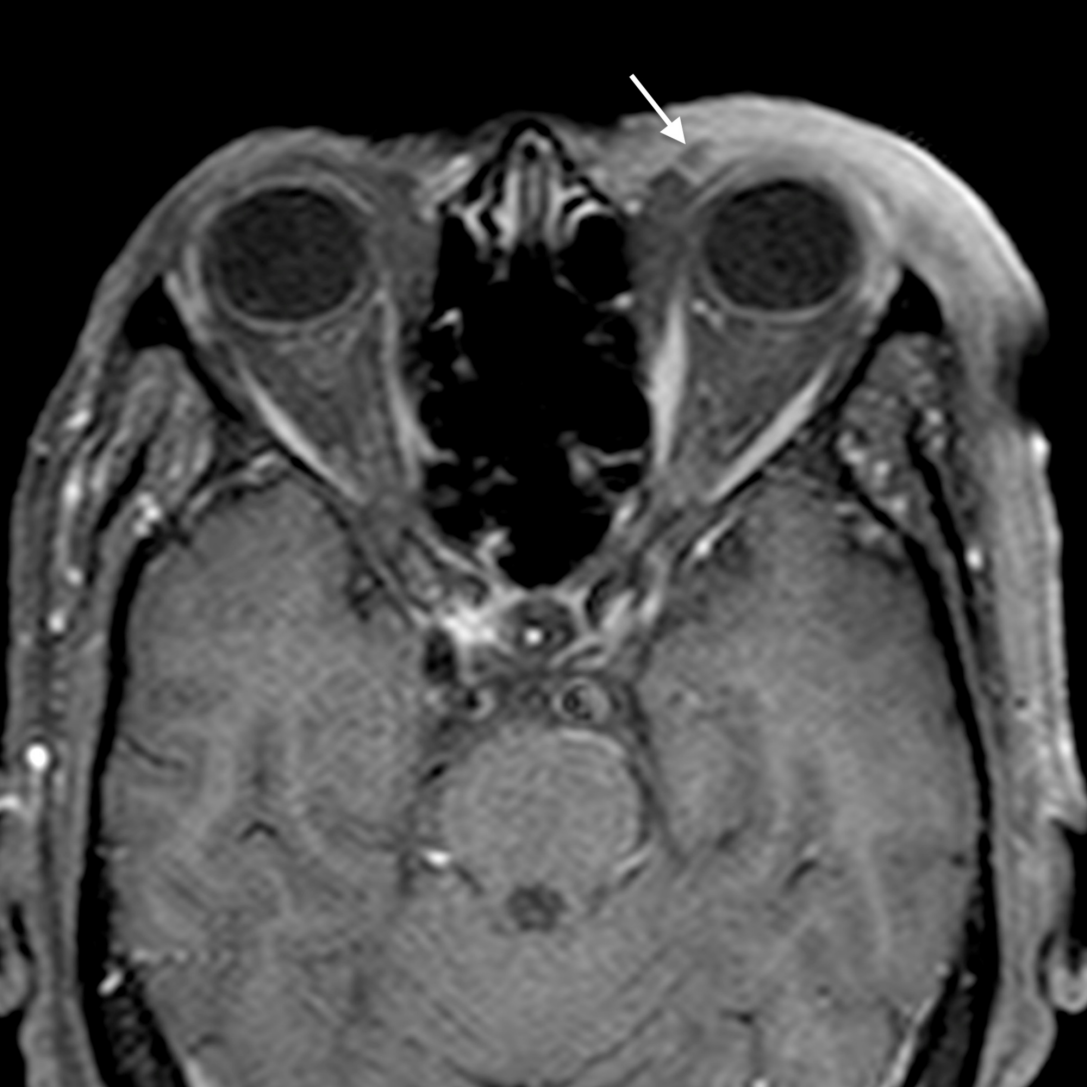
Preseptal cellulitis with abscess: axial post gadolinium T1WI shows diffuse enhancement in the preseptal space with a small peripherally enhancing area medially (arrow), indicative of preseptal abscess T1WI - T1-weighted image

**Figure 9:**
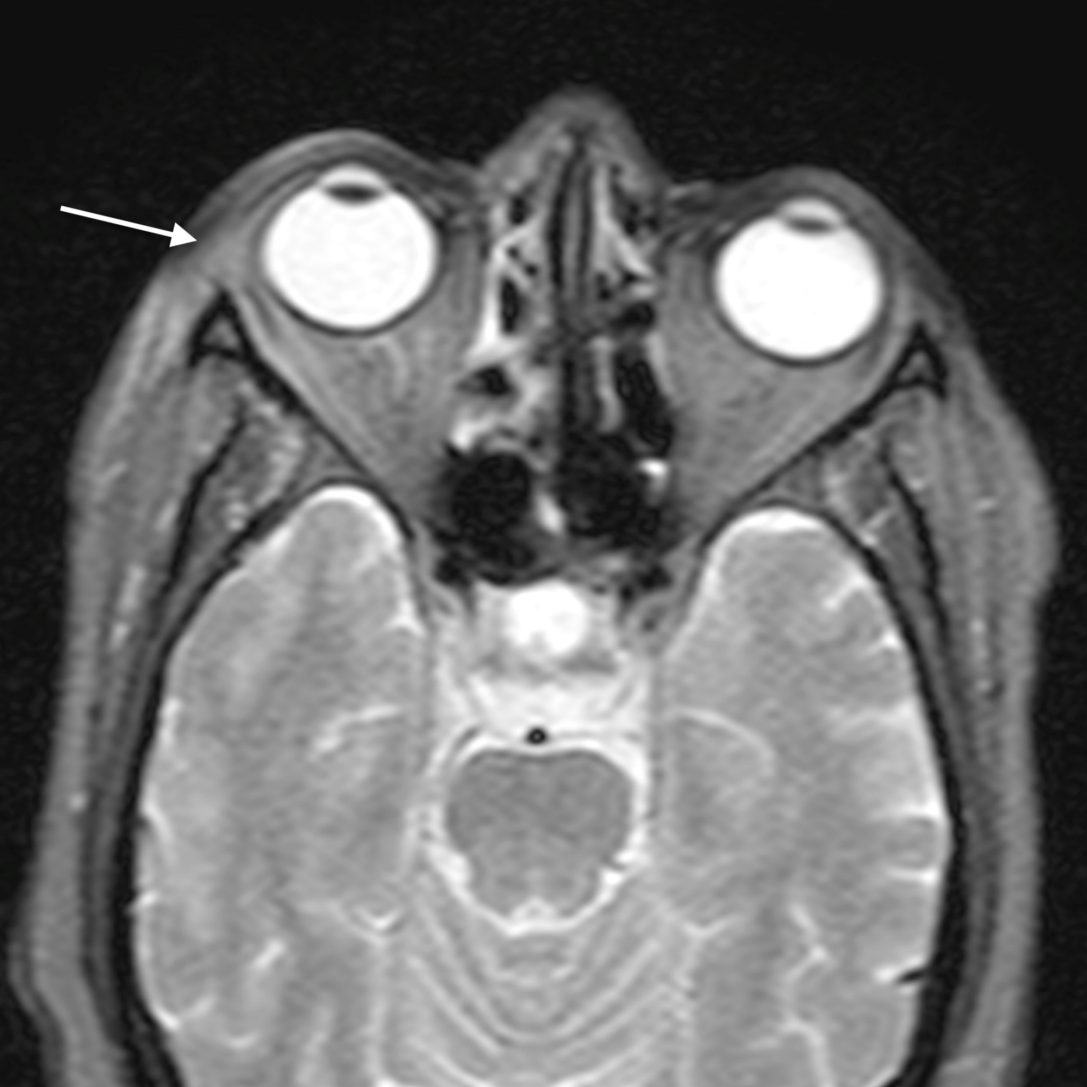
Periorbital cellulitis: axial STIR image shows preseptal soft tissue thickening on right with hyperintense signal (arrow) involving the preseptal region and extending in the infratemporal fossa STIR - short tau inversion recovery

**Figure 10:**
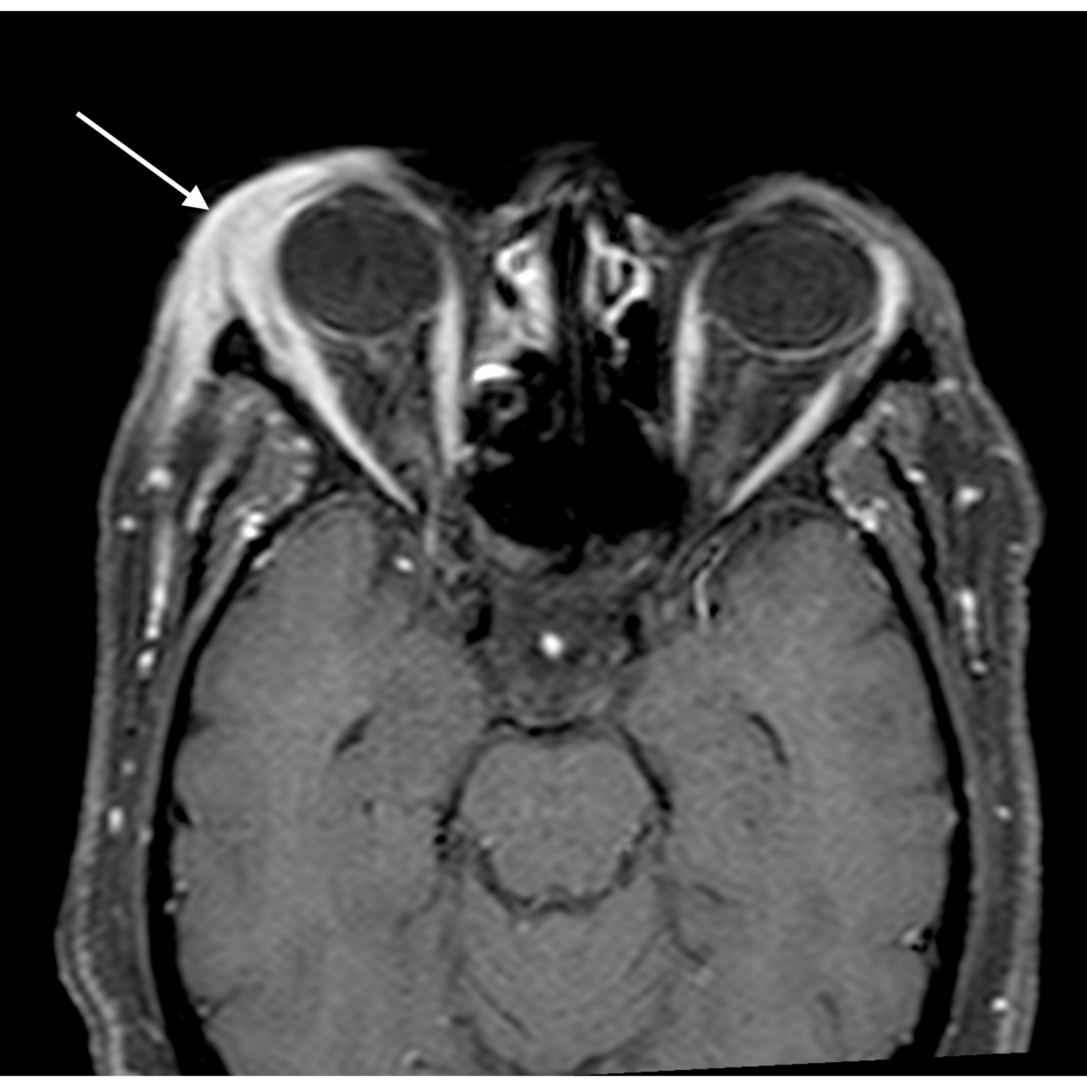
Periorbital cellulitis: axial post gadolinium T1WI shows abnormal enhancement of the preseptal soft tissue thickening (arrow) and extending in the infratemporal fossa T1WI - T1-weighted image

**Figure 11:**
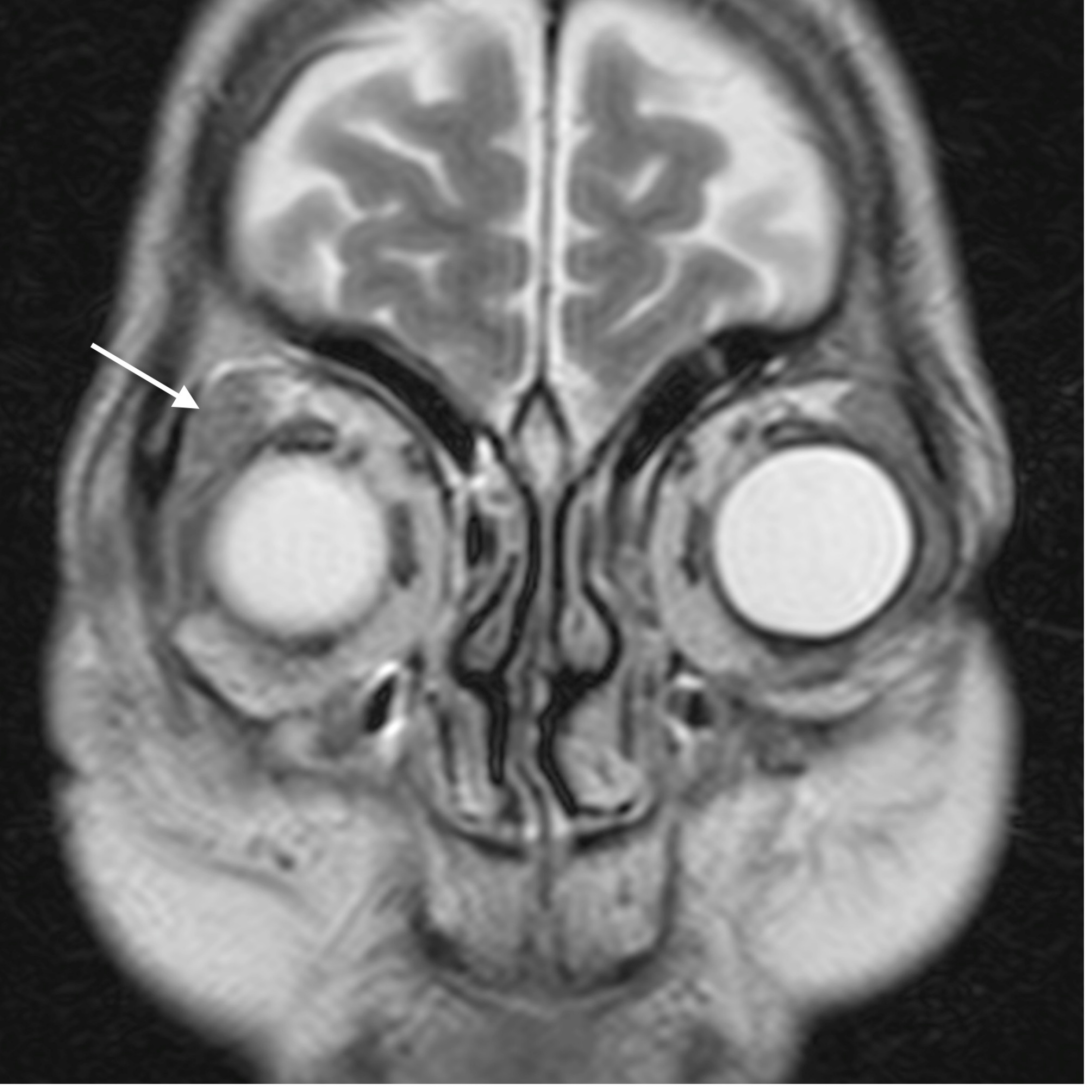
Dacryoadenitis: coronal T2WI shows bulky right lacrimal gland with slight heterogeneous signal (arrow) T2WI - T2-weighted image

**Figure 12:**
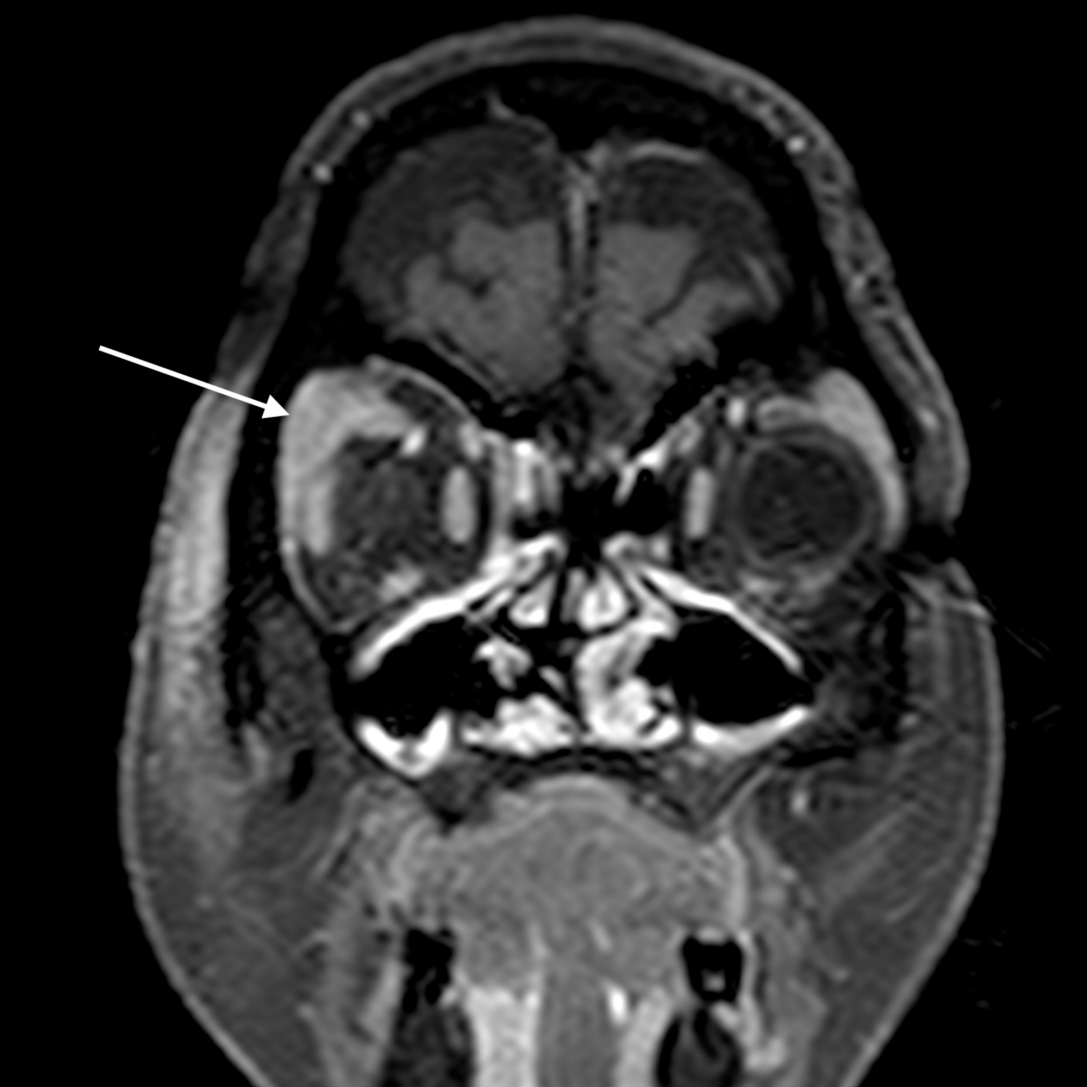
Dacryoadenitis: coronal post gadolinium T1WI shows abnormal enhancement of the enlarged lacrimal gland (arrow) T1WI - T1-weighted image

**Figure 13:**
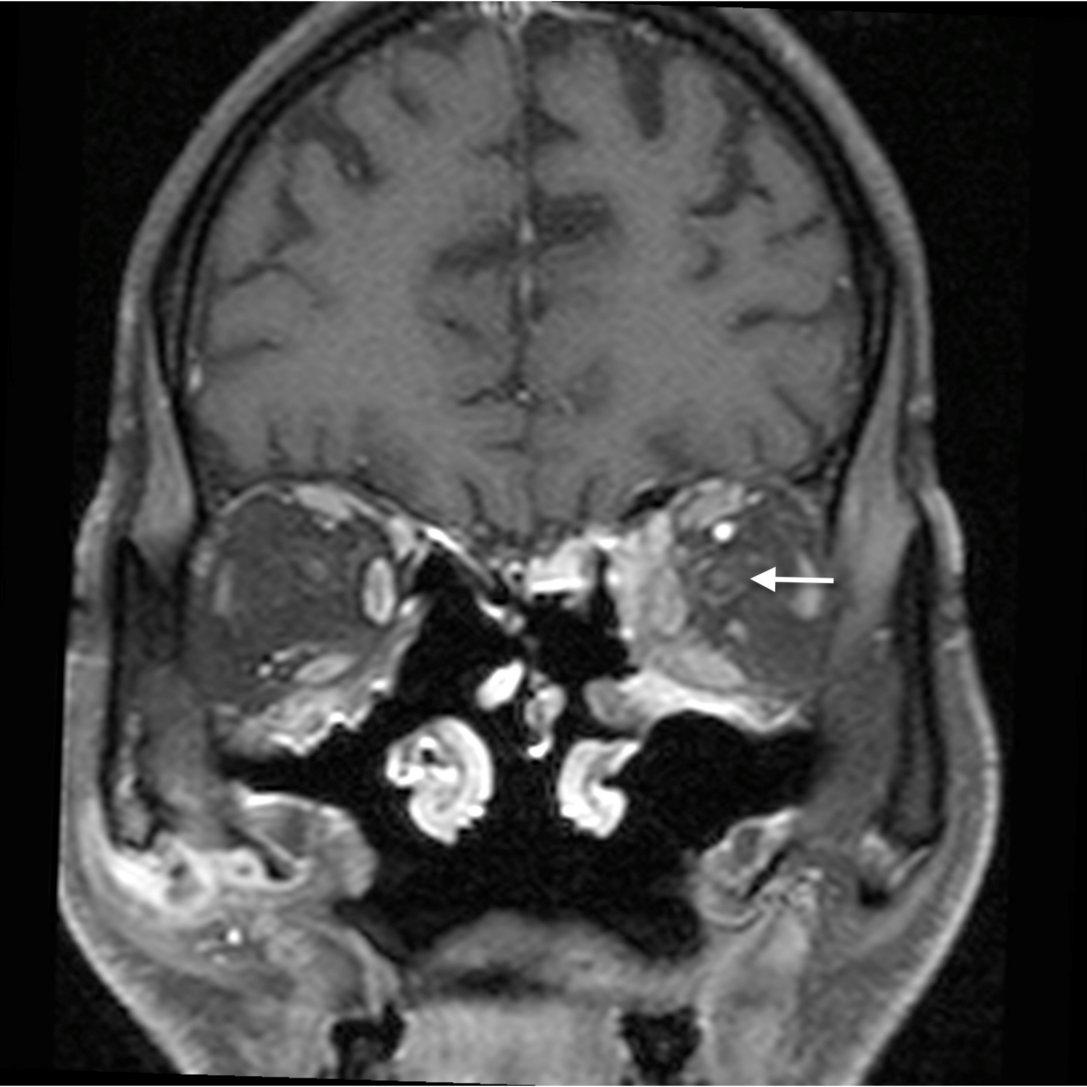
Optic perineuritis: coronal post gadolinium T1WI shows mild perineural enhancement around the left optic nerve (arrow), indicative of optic perineuritis. Enhancement is also noted in the extraconal compartment medially, suggestive of orbital cellulitis T1WI - T1-weighted image

**Figure 14:**
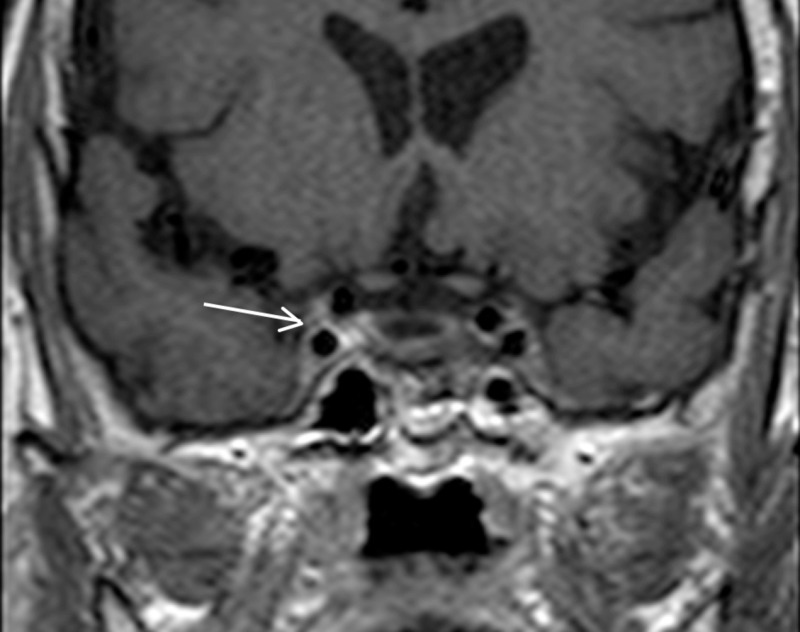
Cavernous sinus thrombophlebitis: coronal post-contrast T1WI (arrow) shows differential enhancement of the right cavernous sinus, suggestive of cavernous sinus thrombophlebitis T1WI - T1-weighted image

The main complications of orbital cellulitis (Figures 2-4) encountered in our study were:

1. An orbital abscess (Figures 5-6);

2. Preseptal cellulitis and abscess (Figures 7-8);

3. Periorbital cellulitis (Figures 9-10);

4. Dacryoadenitis (Figures 11-12);

5. Optic neuritis/perineuritis (Figure 13);

6. Cavernous sinus thrombophlebitis and thrombosis (Figure 14).

The most common complication of orbital cellulitis was orbital/periorbital abscess formation (eight cases, 53.3%), followed by optic neuritis (four cases, 26.67%), intracranial involvement (four cases, 26.67%), dacryoadenitis (three cases, 20%) and cavernous sinus thrombophlebitis (three cases, 20%).

The majority of the patients had more than one symptom, the most common presenting complaint being preseptal edema/swelling, which was present in thirteen patients (86.67%), followed by a diminution of vision, which was present in eight patients (53.3%).

Out of fifteen cases, three patients had dacryoadenitis, eight had orbital/periorbital abscess, four had optic neuritis/perineuritis, four had intracranial involvement, and two patients had bone destruction. Meningitis was found in all the four patients with intracranial involvement, while one of these also had focal cerebritis. One of these cases had pituitary adenoma as an incidental finding. The complications are illustrated in Table 5.

**Table 5:** Complications of orbital cellulitis on MRI * This case had pituitary adenoma as an incidental finding. ** This case had cystic encephalomalacia and acute infarct as an unrelated finding.

Case No.	Dacryoadenitis	Orbital / periorbital abscess	Optic neuritis / perineuritis	Intracranial involvement	Bone destruction
1.	No	No	Yes	No	Yes
2.	No	Yes	No	No	Yes
3.	No	Yes	No	No	No
4.	Yes	Yes	No	No*	No
5.	No	No	No	No**	No
6.	No	No	No	No	No
7.	No	Yes	No	Yes (meningitis)	No
8.	No	Yes	No	No	No
9.	No	Yes	Yes	No	No
10.	Yes	No	No	No	No
11.	No	Yes	No	Yes (focal cerebritis and meningitis)	No
12.	No	No	Yes	No	No
13.	Yes	No	Yes	No	No
14.	No	Yes	No	Yes (meningitis)	No
15.	No	No	No	Yes (meningitis)	No

## Discussion

We studied fifteen cases of orbital cellulitis with special emphasis on the mode of the spread of inflammation from the paranasal sinuses into the adjacent structures, particularly the orbit. The study also evaluated the complications and determined the cause of secondary orbital cellulitis. We found that the most common complication of orbital cellulitis was orbital/periorbital abscess. Other common complications encountered in our study were optic neuritis, dacryoadenitis, cavernous sinus thrombophlebitis, and thrombosis.

In the retrospective study on nine cases of orbital cellulitis and orbital abscess conducted by Sepahdari et al. in 2009, four out of nine cases were in the age-group 51 - 60 years. This is in concordance with our study [9].

Singh et al. in 2017 published a series of four cases of acute orbital cellulitis, which occurred as a complication of rhinosinusitis. They found that the differentiation of preseptal and postseptal orbital cellulitis is clinically difficult [10]. Maxillary sinusitis was found to be the most common cause in their study. One of their cases had both periorbital and orbital cellulitis, while the other three had orbital cellulitis.

The most common predisposing factor for orbital cellulitis is paranasal sinus disease [11]. The most common cause of orbital cellulitis in our study was found to be ethmoid sinusitis, followed by frontal sinusitis. This was concordant with the study done by Chaudhry et al. [12]. However, the disease may also result from infectious processes of the face or pharynx or foreign bodies, or it may develop secondary to trauma or septicemia. Orbital infections arising from secondary to dental infections are rare [13].

Chandler et al. classified the complications of rhinosinusitis, into five groups, based on anatomy and pathogenesis of orbital infection, as 1) inflammatory edema limited to the eyelids and preseptal compartment; 2) orbital cellulitis; 3) subperiosteal abscess; 4) orbital abscess, and 5) cavernous sinus thrombosis [14].

The factor primarily responsible for the development of orbital cellulitis in ethmoid sinusitis is the anatomic contiguity of the two structures, thin lamina papyracea and the valveless ethmoidal veins, which allow rapid spread of infection into the orbit [14]. Apart from being thin, the lamina papyracea has multiple tiny natural deficiencies, perforating nerve vessels [15]. Another common method of spread of infection is by means of interference with the venous drainage of orbital contents. Infection from the nasal cavity and paranasal sinuses may also spread by means of myriad direct connections of the veins of these structures with the veins of orbit and cavernous sinuses [14]. In the absence of drainage, an orbital abscess carries a high risk of intracranial spread and visual impairment [16-17]. Thus, it is imperative to drain an orbital or subperiosteal abscess without any delay. In addition, without drainage of the abscess, the antibacterial potency of the antibiotics is reduced because of inadequate enzymatic degradation of the bacteria in the purulent milieu [18].

The periorbita is the periosteum lines the walls of the bony orbit and it serves as a barrier against the early spread of infection from the paranasal sinus into the orbit. Therefore, disease spread from the ethmoid sinus to the orbit initially leads to subperiosteal abscess formation before it spreads into the orbit [19]. The subperiosteal orbital abscess may expand rapidly and cause more serious complications like orbital and cerebral abscesses [20]. Intracranial complications of orbital cellulitis are usually catastrophic unless promptly treated. Orbital infection can extend into the subdural space, leading to subdural empyema. Progression of the disease can cause orbital vein thrombosis, cavernous sinus thrombosis, and rarely even mycotic aneurysm of the internal carotid artery. Other known intracranial complications are epidural empyema, cerebritis, brain abscess, and meningitis. If intracranial complications occur as a result of sinusitis, functional endoscopic sinus surgery is required. In the presence of empyema and brain abscess, neurosurgical intervention may be necessary. In addition, frontal sinusitis can directly spread to the anterior cranial fossa via bone dehiscence or when frontal bone osteomyelitis develops [21].

To optimize image resolution in orbital MRI, phased-array surface coils should be used to increase the signal to noise ratio (SNR), though we used volume coils in our study and got sufficiently good quality images. Due to the small field of view of the examination area, the improvement in spatial resolution and soft tissue contrast leads to an increase in image quality. Unenhanced axial T1-weighted images display anatomic relationships and can detect lesions embedded within fat. Gadolinium (Gd)-enhanced images improve the delineation of margins in many lesions. Fat-suppression techniques, such as short tau inversion recovery and frequency-selected fat suppression, may improve the conspicuity of soft-tissue lesions embedded in fatty tissue by selectively diminishing the hyperintensity of fat on T1-weighted images. Field inhomogeneity and artifacts may partially diminish the potential advantages of this technique.

## Conclusions

Our study highlights the role of MRI in evaluating the spectrum of orbital cellulitis and its complications. MRI is better suited to stage orbital cellulitis and determine its pattern of spread due to its higher contrast resolution than CT. It is vital in assessing the extent of the orbital infection, underlying paranasal sinus involvement as well as in the detection of complications of orbital cellulitis, especially intracranial spread. There has been no large case series of MRI findings in orbital cellulitis, published as per our knowledge. Our study is probably the first large case series on the topic.
